# Association of acute kidney injury with readmissions after hospitalization for acute exacerbation of chronic obstructive pulmonary disease: a population-based study

**DOI:** 10.1186/s12882-020-01780-2

**Published:** 2020-04-03

**Authors:** Atsushi Hirayama, Tadahiro Goto, Kohei Hasegawa

**Affiliations:** 1 Department of Emergency Medicine, Massachusetts General Hospital, Boston, MA, 125 Nashua Street, Suite 920, Boston, MA USA; 2 Public Health, Department of Social Medicine, Osaka University Graduate School of Medicine, 2-2, Yamadaoka, Suita, Osaka, Japan; 3 Department of Clinical Epidemiology and Health Economics, School of Public Health, The University of Tokyo, 7-3-1, Hongo, Bunkyo-ku, Tokyo, Japan

**Keywords:** Acute exacerbation of COPD, Acute kidney injury, Readmission, Population-based study

## Abstract

**Background:**

Little is known about the relationship between acute kidney injury (AKI) and outcomes after acute exacerbation of chronic obstructive pulmonary disease (AECOPD). We aimed to investigate associations between AKI and readmission risks after hospitalization for AECOPD.

**Methods:**

A retrospective, population-based cohort study using State Inpatient Databases from seven U.S. states (Arkansas, California, Florida, Iowa, Nebraska, New York, and Utah) from 2010 through 2013. We identified all adults (aged ≥40 years) hospitalized for AECOPD during the study period. Among them, we further identified patients with a concurrent diagnosis of new AKI. The outcome measures were any-cause readmissions within 30 days and 90 days after hospitalization for AECOPD. To determine associations between AKI and readmission risk, we constructed Cox proportional hazards models examining the time-to-readmission. We also identified the primary reason of readmission.

**Results:**

We identified 356,990 patients hospitalized for AECOPD. The median age was 71 years and 41.9% were male. Of these, 24,833 (7.0%) had a concurrent diagnosis of AKI. Overall, patients with AKI had significantly higher risk of 30-day all-cause readmission compared to those without AKI (hazard ratio 1.47; 95% CI 1.43–1.51; *P* < 0.001). Likewise, patients with AKI had significantly higher risk of 90-day all-cause readmission (hazard ratio 1.35; 95% CI 1.32–1.38; *P* < 0.001). These associations remained significant after adjustment for confounders (both *P* < 0.05). Additionally, patients with AKI were likely to be readmitted for non-respiratory reasons including sepsis, acute renal failure, and congestive heart failure.

**Conclusions:**

Among patients hospitalized for AECOPD, patients with AKI were at higher risk of 30-day and 90-day readmission, particularly with non-respiratory reasons.

## Background

Acute exacerbation of chronic obstructive pulmonary disease (AECOPD) is a major public health burden accounting for 600,000 hospitalizations in the US annually [1]. As a readmission after hospitalization for AECOPD is a common and costly event [2, 3], investigating its characteristics and risks are important. Acute kidney injury (AKI) is another important public health burden; the incidence of AKI has been reported to be 5% of hospitalized patients and 30% of critically ill patients [4]. These two acute conditions share similar pathobiology in the development and progression, such as systemic inflammation [5].

The literature has reported relationships between these two conditions. For example, according a study using clinical database of the United Kingdom, the incidence and prevalence of AKI were higher in patients with COPD compared to those reported in previous studies in general populations and hospitalizations [6]. Additionally, cross-sectional studies have also reported that patients hospitalized for AECOPD with AKI had higher in-hospital mortality, compared to those without AKI [6, 7]. Despite the clinical importance, no longitudinal study has investigated whether AKI is associated with the subsequent risk of readmissions in patients hospitalized for AECOPD—a population with large morbidity burden and healthcare use.

To address this knowledge gap, we aimed to investigate the association of AKI with all-cause readmissions within 30 and 90 days after hospitalization for AECOPD.

## Methods

### Study design and setting

We conducted a retrospective cohort study using large, population-based data from the Healthcare Cost and Utilization Project (HCUP) State Inpatient Database (SID) of seven geographically-dispersed US states (Arkansas, California, Florida, Iowa, Nebraska, New York, and Utah) from 2010 through 2013. The HCUP is a family of healthcare databases that are developed through a federal-state-industry partnership and sponsored by the Agency for Healthcare Research and Quality (AHRQ). The HCUP is the largest collection of longitudinal hospital care data in the US, with all-payer, encounter-level information. The HCUP SIDs capture *all* hospitalizations, regardless of source of disposition, from acute care, non-federal, general and other specialty hospitals within the participating states [8]. These seven states were selected for their high data quality, geographic distribution, and mainly because their data included unique encrypted patient identifiers that enable longitudinal follow-up of specific individuals across years (including identification of readmissions). The institutional review board of Massachusetts General Hospital approved this study.

### Study population

We identified all hospitalized adult patients (aged > 40 years) with a principal discharge diagnosis of COPD, as defined by the *International Classification of Diseases, Ninth Revision, Clinical Modification* (*ICD-9-CM*) diagnosis codes of 491.21, 491.22, 491.8, 491.9, 492.8, 493.20, 493.21, 493.22, and 496, or those with a primary diagnosis of respiratory failure (codes 518.81, 518.82, 518.84, and 799.1) and a secondary diagnosis of COPD [9, 10]. In the current analysis, we used only the first hospitalizations of the eligible patients during the study period. We also excluded patients who left the hospital against medical advice, those who died in-hospital at their index hospitalization, those who were transferred to another acute-care facility, and out-of-state residents.

### Measurements

The SID contain information on the patient characteristics, including demographics (age, sex, and race/ethnicity), primary insurance type (payer), quartiles for estimated household income, patient residence, *ICD-9-CM* diagnosis and procedure codes, patient comorbidities (29 Elixhauser comorbidity measures and arrhythmia), hospital course (e.g., hospital length-of-stay, in-hospital death), and disposition.

### Primary exposure

The primary exposure was the development of in-hospital AKI during the index hospitalization for AECOPD, as defined by the *ICD-9-CM* diagnostic codes of 584.5, 584.6, 584.7, 584.8, and 584.9 in any diagnostic fields [11–13], with excluding AKI as an admission diagnosis. Additionally, we also identified AKI with the use of dialysis, defined as having both of AKI (diagnostic codes, 584.5–584.9) and hemodialysis (procedure code of 39.95 or diagnostic code of V45.1, V56.0 or V56.1) [11, 12].

### Outcome measures

The outcome measures were readmission attributable to any cause within 30 and 90 days of discharge from the index hospitalization for AECOPD. In the COPD literature, 30-day readmission rates have been investigated [9, 13, 14] in the context of the *Centers for Medicare and Medicaid Services’* Hospital Readmissions Reduction Program (HRRP) [15]; 90-day readmission rates have also been recognized as an important clinical indicator [16, 17]. The secondary outcome measure was the primary discharge diagnosis of the readmission. To make data presentation and interpretation more meaningful, we consolidated the principal discharge diagnoses (> 14,000 *ICD-9-CM* diagnosis codes) into 285 mutually exclusive diagnostic categories by using the AHRQ-defined *Clinical Classifications Software* (*CCS*) [18].

### Statistical analysis

First, we compared the patient characteristics between patients with and without AKI by using Wilcoxon rank sum test or chi-squared test, as appropriate. We also compared Kaplan-Meier curves between the two groups with the use of the log-rank test. Next, we modeled the time-to-readmission by fitting Cox proportional hazards models with generalized estimating equations accounting for patient clustering within hospitals (e.g., severity of patients, physicians’ preference in disease management within hospitals) [19, 20]. The time-to-readmission for each patient was defined as the period from the discharge to when the first readmission occurred within the 30-day and 90-day follow-up periods. Patients who did not have an outcome were censored at 30 days (or 90 days) from discharge or in-hospital death during the corresponding follow-up period, whichever occurred first. We fitted Cox proportional hazards model with adjustment for potential confounders, such as age, sex, race/ethnicity, primary insurance, quartiles for median household income, residential status, length-of-stay at the index hospitalization, hospital state, and 28 Elixhauser comorbidities as well as arrhythmia [21, 22]. Furthermore, as sensitivity analyses, we repeated the analysis with stratifications by age category (40–64 years and ≥ 65 years) and sex as previous studies have reported age- and sex-related differences in the readmission rate after hospitalization for AECOPD [9, 13, 14]. Additionally, we repeated the analysis with stratifications by presence of renal failure (or chronic kidney disease [CKD]) indicated in the Elixhauser comorbidities. Lastly, we compared the 30-day and 90-day readmission rates and calculated the unadjusted and adjusted hazard ratios among patients without AKI, those with AKI without dialysis use, and healthcare use and with AKI and dialysis use. We primarily conducted an available case analysis, and examined consistency with the results of complete case analysis. All analyses used STATA 14.0 (STATA Corp, College Station, TX). All *P* values were two-tailed, with *P* < 0.05 considered statistically significant.

## Results

We first identified 385,604 patients hospitalized for AECOPD in the seven U.S. states. From these, we excluded 6911 patients who left the hospital against medical advice, 5613 patients who died in-hospital at the index hospitalization, 4378 patients who were transferred to another acute-care facility, and 11,712 out-of-state residents. Finally, a total of 356,990 patients were eligible for the present analysis (Supplemental Figure 1). Overall, the median age was 71 years, 41.9% were male, and 73.9% were non-Hispanic white; 7.0% had a new diagnosis of AKI during the index hospitalization. The patient characteristics differed between patients with AKI and those without AKI (Table 1)—for example, the AKI group was more likely to be older and male. Overall, 58,076 (16.3%) patients had at least one readmission within 30 days after their index hospitalization, and 112,917 (31.6%) had at least one readmission within 90 days after their hospitalization (Supplemental Table 1).

**Table 1:** Characteristics of patients hospitalized for acute exacerbation of chronic obstructive pulmonary disease by acute kidney injury

Characteristics	AKI	Non-AKI	***P*** value
	*n* = 24,833 (7.0%)	*n* = 332,157 (93.0%)	
Age (year), median (IQR)	76 (67–83)	70 (60–80)	< 0.001
Male	12,605 (50.8)	136,949 (41.2)	< 0.001
Race/ethnicity			< 0.001
Non-Hispanic white	17,375 (72.3)	237,741 (74.0)	
Non-Hispanic black	2848 (11.9)	34,030(10.6)	
Hispanic	2593 (10.8)	35,333(11.0)	
Others	1217 (5.1)	14,034 (4.5)	
Primary health insurance			< 0.001
Medicare	20,089 (81.4)	233,472 (70.3)	
Medicaid	1817 (7.4)	36,878 (11.1)	
Private	1682 (6.8)	38,944 (11.7)	
Others	1093 (4.4)	22,698 (6.9)	
Median household income quartile			< 0.001
1 (lowest)	7216 (29.7)	103,211 (31.9)	
2	6230 (25.6)	89,605 (27.7)	
3	5931 (24.4)	76,312 (23.6)	
4 (highest)	4927 (20.3)	54,794 (16.9)	
Patient residence			< 0.001
Metropolitan	22,529 (90.8)	288,858 (87.0)	
Non-metropolitan	2304 (9.2)	43,299 (13.0)	
Selected comorbidities^a^			
Congestive heart failure	11,326 (45.6)	78,120 (23.5)	< 0.001
Depression	3348 (13.5)	52,482 (15.8)	< 0.001
Diabetes	10,415 (41.9)	93,904 (28.3)	< 0.001
Hypertension	20,125 (81.0)	219,396 (66.1)	< 0.001
Obesity	5048 (20.3)	50,795 (15.3)	< 0.001
Peripheral artery disease	2967 (11.9)	24,961 (7.5%)	< 0.001
Chronic kidney disease	13,481 (54.3)	34,518 (10.4)	< 0.001
Hospital length-of-stay			< 0.001
< 3 days	3599 (14.5)	97,877 (29.5)	
3–4 days	7223 (29.1)	117,624 (35.4)	
5–6 days	5407 (21.8)	60,523 (18.2)	
≥ 7 days	8604 (34.6)	56,133 (16.9)	
Hospital state			< 0.001
Arkansas	1695 (6.8)	15,644 (4.7)	
California	4561 (18.4)	63,913 (19.2)	
Florida	10,295 (41.5)	135,717 (40.9)	
Iowa	764 (3.1)	11,825 (3.6)	
Nebraska	536 (2.2)	7252 (2.2)	
New York	6667 (26.8)	93,863 (28.3)	
Utah	315 (1.3)	3943 (1.2)	

*Abbreviations*: *AKI* Acute kidney injury, *IQR* Interquartile range

Data are shown as n (%) unless otherwise specified

^a^Selected from 29 Elixhauser comorbidity measures and arrhythmia

**Fig. 1:**
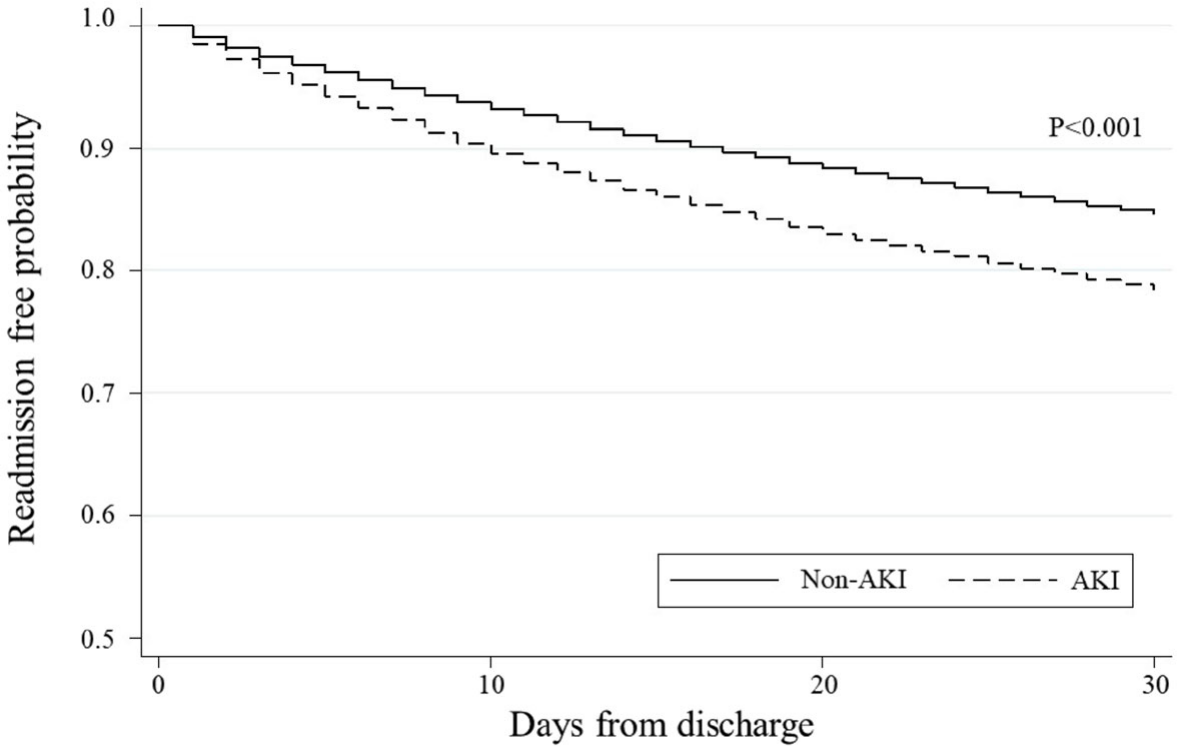
Kaplan-Meier survival estimates of all-cause readmission risk during 30-day period after the index hospitalization for acute exacerbation of chronic obstructive pulmonary disease. Patients with acute kidney injury (AKI) had a significantly higher risk of all-cause readmission during 30-day period after the index hospitalization, compared to those without AKI (P_log-rank_ < 0.001)

**Fig. 2:**
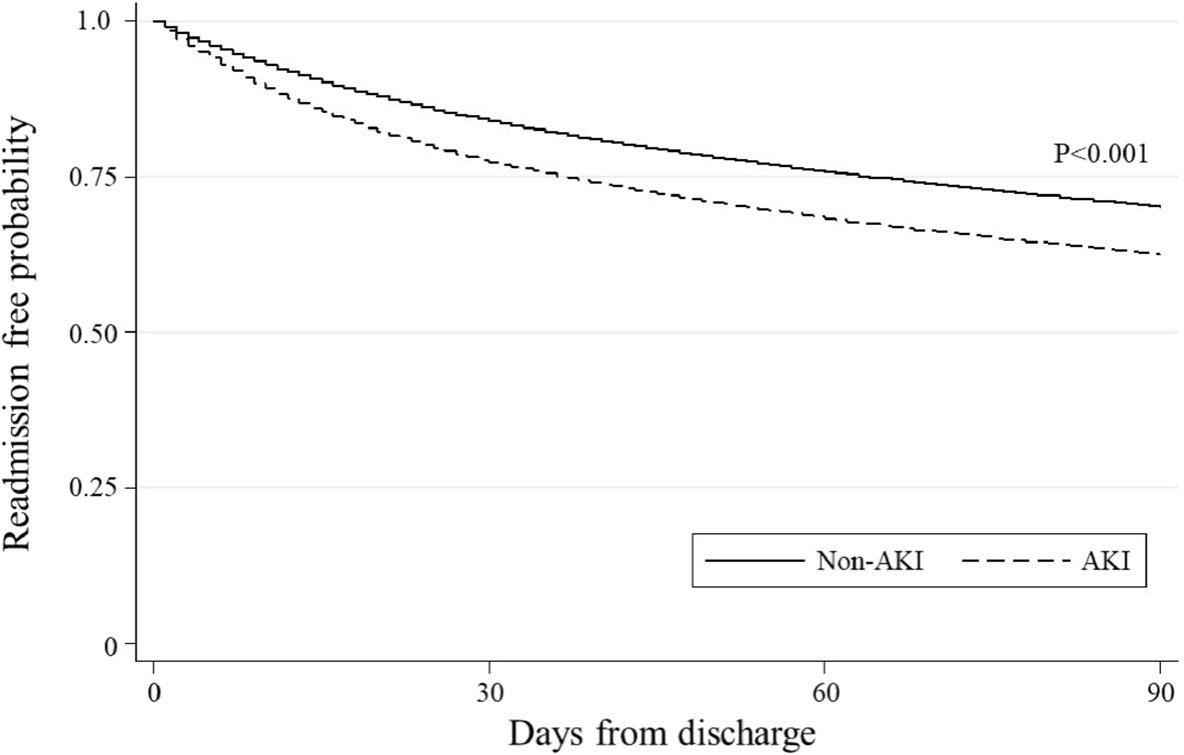
Kaplan-Meier survival estimates of all-cause readmission risk during 90-day period after the index hospitalization for acute exacerbation of chronic obstructive pulmonary disease. Patients with acute kidney injury (AKI) had a significantly higher risk of all-cause readmission during 90-day period after the index hospitalization, compared to those without AKI (P_log-rank_ < 0.001)

The Kaplan-Meier survival curves demonstrated a significant difference in the risk of all-cause 30-day and 90-day readmission between patients with AKI and those without AKI (*P* < 0.001, Figs. 1 and 2). In the unadjusted Cox proportional hazards model, patients with AKI had a significantly higher risk of 30-day readmission when compared to those without AKI (hazard ratio [HR] 1.47; 95%CI 1.43–1.51; *P* < 0.001; Table 2). In the adjusted model, the significant association persisted (HR 1.07; 95%CI 1.04–1.11; *P* < 0.001). Likewise, with regard to all-cause 90-day readmission, patients with AKI had a significantly higher risk of 90-day readmission (HR 1.35; 95%CI 1.32–1.38; *P* < 0.001; Table 3). The significant association also persisted after adjustment for potential confounders (HR 1.03; 95%CI 1.00–1.05; *P* = 0.04). With stratifications by age, sex and presence of CKD indicated in the Elixhauser comorbidities, these associations remained significant in patients aged ≥65 years, women, and patients without CKD (all *P* < 0.05, Tables 2 and 3). There was a statistically significant interaction between AKI and the presence of CKD on the risks of 30-day and 90-day readmission after hospitalization for AECOPD (both P_interaction_ < 0.05). The complete case analyses also showed consistent results (Supplemental Tables 2, 3 and 4). In the stratified analysis by dialysis use, both AKI groups had higher 30-day and 90-day readmission rates, compared with those without AKI (unadjusted *P* < 0.001; Supplemental Table 5). Despite the limited statistical power in this stratified analysis, the association between AKI and higher 30-day readmission rate remained significant after adjustment in the AKI without hemodialysis group.

**Table 2:** Hazard ratio for all-cause readmission during 30-day periods after index hospitalization for acute exacerbation of chronic obstructive pulmonary disease, according to acute kidney injury, overall and stratified by age category and sex and chronic kidney disease

	30-day readmission rate		Unadjusted model		Adjusted model^**a**^		P for interaction
	AKI (*n* = 24,833)	Non-AKI (*n* = 332,157)	HR (95% CI)	*P*-value	HR (95% CI)	*P*-value	
**Overall** (*n* = 356,990)	22.4%	15.8%	1.47 (1.43–1.51)	< 0.001	1.07 (1.04–1.11)	< 0.001	
**Age category**							
40–64 years (*n* = 122,362)	20.9%	14.0%	1.52 (1.43–1.63)	< 0.001	1.03 (0.96–1.10)	0.45	0.13
≥ 65 years (*n* = 234,628)	22.7%	16.8%	1.41 (1.36–1.45)	< 0.001	1.09 (1.05–1.13)	< 0.001	
**Sex**^b^							
Men (*n* = 149,333)	22.0%	16.7%	1.36 (1.30–1.41)	< 0.001	1.03 (0.99–1.08)	0.14	0.07
Women (*n* = 207,436)	22.7%	15.2%	1.56 (1.50–1.62)	< 0.001	1.12 (1.07–1.17)	< 0.001	
**Chronic kidney disease**^c^							
Chronic kidney disease (*n* = 47,999)	27.7%	24.2%	1.14 (1.09–1.18)	< 0.001	1.03 (0.99–1.07)	0.24	0.01
No chronic kidney disease (*n* = 308,991)	22.3%	16.8%	1.32 (1.26–1.38)	< 0.001	1.09 (1.05–1.15)	< 0.001	

*Abbreviations*: *AKI* Acute kidney injury, *HR* Hazard ratio, *CI* Confidence interval

^a^Cox proportional hazards model adjusting for age, sex, race/ethnicity, insurance status, estimated household income, residential status, hospital length-of-stay, hospital state, and Elixhauser comorbidity measures and arrhythmia with generalized estimating equations to account for patient clustering within hospitals

^b^221 patients with missingness on sex data

^c^Chronic kidney disease was defined by the Elixhauser comorbidities

**Table 3:** Hazard ratio for all-cause readmission during 90-day periods after index hospitalization for acute exacerbation of chronic obstructive pulmonary disease, according to acute kidney injury, overall and stratified by age category and sex and chronic kidney disease

	90-day Readmission rate		Unadjusted model		Adjusted model^**a**^		P for interaction
	AKI (*n* = 24,833)	Non-AKI (*n* = 332,157)	HR (95% CI)	*P*-value	HR (95% CI)	*P*-value	
**Overall** (*n* = 356,990)	39.7%	31.0%	1.35 (1.32–1.38)	< 0.001	1.03 (1.00–1.05)	0.04	
**Age category**							
40–64 years (*n* = 122,362)	37.6%	27.8%	1.43 (1.36–1.50)	< 0.001	1.00 (0.95–1.06)	0.86	0.10
≥ 65 years (*n* = 234,628)	40.2%	32.8%	1.29 (1.26–1.32)	< 0.001	1.03 (1.00–1.06)	0.03	
**Sex**^b^							
Men (*n* = 149,333)	38,9%	32.2%	1.26 (1.22–1.29)	< 0.001	0.99 (0.96–1.03)	0.59	0.06
Women (*n* = 207,436)	40.5%	30.2%	1.43 (1.39–1.48)	< 0.001	1.06 (1.02–1.09)	0.001	
**Chronic kidney disease**^c^							
Chronic kidney disease (*n* = 47,999)	43.0%	40.9%	1.06 (1.03–1.09)	0.001	0.98 (0.95–1.02)	0.21	< 0.001
No chronic kidney disease (*n* = 308,991)	35.7%	29.9%	1.22 (1.18–1.26)	< 0.001	1.04 (1.00–1.07)	0.04	

*Abbreviations*: *AKI* Acute kidney injury, *HR* Hazard ratio, *CI* Confidence interval

^a^Cox proportional hazards model adjusting age, sex, race/ethnicity, insurance status, estimated household income, residential status, hospital length-of-stay, hospital state, and Elixhauser comorbidity measures and arrhythmia with generalized estimating equations to account for patient clustering within hospitals

^b^221 patients with missingness on sex data

^c^Chronic kidney disease was defined by the Elixhauser comorbidities

Among patients without AKI, the most frequent primary cause for 30-day readmission was COPD, followed by pneumonia, respiratory failure, and asthma; Table 4). By contrast, patients with AKI were more likely to be readmitted due to non-respiratory diseases (e.g., sepsis, acute renal failure, congestive heart failure). The five most frequent causes of readmission were not different between 30-day and 90-day readmissions after hospitalization for AECOPD (Table 4 and Supplemental Table 6).

**Table 4:** The ten most frequent principal diagnoses of 30-day readmissions in patient hospitalized for acute exacerbation of chronic obstructive pulmonary disease, stratified by acute kidney injury

Without AKI (***n*** = 50,984)		With AKI (***n*** = 8151)	
Primary diagnosis^**a**^	n (%)	Primary diagnosis^**a**^	n (%)
COPD and bronchiectasis	12,954 (25.4)	Septicemia	1176 (14.4)
Pneumonia	3947 (7.7)	Acute renal failure	963 (11.8)
Respiratory failure	3571 (7.0)	COPD and bronchiectasis	944 (11.6)
Asthma	3255 (6.4)	Congestive heart failure	713 (8.8)
Congestive heart failure	3.019 (5.9)	Respiratory failure	623 (7.6)
Septicemia	1806 (3.5)	Pneumonia	579 (7.1)
Cardiac dysrhythmia	1395 (2.7)	Asthma	204 (2.5)
Nonspecific chest pain	921 (1.8)	Acute myocardial infarction	166 (2.0)
Aspiration pneumonitis	683 (1.3)	Nonspecific chest pain	166 (2.0)
Fluid and electrolyte disorders	666 (1.3)	Intestinal infection	158 (1.9)

^a^The primary diagnosis codes (> 14,000 *ICD-9-CM* diagnosis codes) are consolidated into 285 mutually exclusive diagnostic categories by using the AHRQ-defined *Clinical Classifications Software*

## Discussion

In this large population-based study of 356,990 patients hospitalized for AECOPD in the seven U.S. states, we found that patients with AKI had a 50% higher risk of all-cause readmissions during 30 days after their index hospitalization when compared to those without AKI. This significant association persisted after the adjustment for potential confounders. The limited epidemiological literature has indicated the association of acute kidney disease with COPD [23]. For example, a retrospective cohort study using electronic medical records in Taiwan showed that COPD is associated with a higher risk of development of AKI [23]. Furthermore, among patients hospitalized for AECOPD, those with AKI had approximately 2-fold higher mortality rate within 6 months compared to those without AKI [6]. Our findings based on the large population-based data corroborate these prior studies, and extend them by demonstrating the longitudinal association of AKI with a higher risk of all-cause readmission after hospitalization for AECOPD.

The underlying mechanisms of the observed association of AKI with higher risks of readmission after hospitalization for AECOPD warrant clarification. In the present study, part of the association was attributable to the differences in patient-level socio-demographic factors, hospital length-of-stay and comorbidities between the two target populations. However, despite the rigorous adjustment, AKI remained an independent risk factor for readmission in patients hospitalized for AECOPD. This finding suggests that there are other factors predisposing patients with AKI to worse clinical outcomes. One potential mechanism is impaired immunity following AKI [24, 25]. Indeed, studies have showed that AKI is a risk factor for subsequent infection (e.g., incident active tuberculosis infection [26] and sepsis [27]). Consistently, we observed that the most frequent primary cause of readmission was sepsis in the patients with AKI, while the most common cause was COPD in those without AKI. Furthermore, AKI-related systemic inflammation [5] and volume overload [28] may add to the existent morbidity in patients hospitalized for AECOPD. These potential mechanisms may have independently or jointly contributed to worse disease control of COPD, exacerbation of existent comorbidities, and increased disease susceptibility, thereby leading to greater healthcare utilization in patients with concurrent AECOPD and AKI. Additionally, we also observed the age- and sex-related difference in the association of AKI with readmission risks after hospitalization for AECOPD. Consistently, previous epidemiologic studies have also indicated the age and sex-related difference in severity of AKI, morbidity and mortality after AKI in other disease populations [6, 12, 29–33]. These data collectively suggest the interplay between the patient biological characteristics (age, sex), AKI, and AECOPD, and their integrated contributions to subsequent morbidity risk, which merits further investigations.

Our study has several potential limitations. First, in this large population-based study, detailed information on renal function was unavailable. Yet, adjusting for the Elixhauser comorbidities should have accounted, at least partially, for potential confounding by this factor. Additionally, to account for the potential effect of AKI severity, we also performed the sensitivity analysis stratified by use of dialysis. Second, as we used administrative datasets, there may be misclassifications, such as misdiagnosis of AECOPD and AKI. Nevertheless, the *ICD-9-CM* codes for COPD are widely used [8, 9], and the HCUP data are rigorously tested and considered accurate [13, 34–36]. Additionally, while the literature showed that the *ICD-9-CM* codes for AKI had a high specificity and low sensitivity [37], underdiagnoses or misclassifications of AKI at the index hospitalization are likely to have occurred equally regardless of the subsequent outcomes, which would have biased our estimates toward the null. Third, SIDs do not capture information on out-of-hospital deaths, which precluded us from accounting for this potential competing risk. In contrast, we accounted for the potential effect of in-hospital deaths during the follow-up periods. Fourth, as with any observational study, the causal inference of AKI with readmission risks might be confounded by unmeasured factors (e.g., access to ambulatory healthcare, patient’s health behavior). Lastly, the studied data are not a random sample of all individuals with AECOPD in the U.S. However, the seven geographically-dispersed states represent approximately 27% of the U.S. population, thereby supporting the generalizability of our inferences.

## Conclusions

In the large population-based database of 356,990 patients hospitalized for AECOPD across seven US states, we found that patients with AKI had a significantly higher risk of all-cause readmissions during 30 and 90 days after their index hospitalization, compared to those without AKI. For clinicians, our findings underscore the importance of prevention of AKI and post-discharge care in this population. For researchers, our findings should facilitate further investigations into the mechanisms underlying the COPD-AKI link to develop preventive and therapeutic interventions in this population with large morbidity burden and healthcare use.

## Supplementary information

**Additional file 1 **: **Figure S1**. Patient flow of the study. AECOPD, acute exacerbation of chronic obstructive pulmonary disease.

**Additional file 2 **: **Table S1**. Characteristics of patients hospitalized for acute exacerbation of chronic obstructive pulmonary disease by readmission status. **Table S2**. Characteristics of patients hospitalized for acute exacerbation of chronic obstructive pulmonary disease by acute kidney injury (complete case analysis). **Table S3**. Hazard ratio for all-cause readmission during 30-day periods after index hospitalization for acute exacerbation of chronic obstructive pulmonary disease, according to acute kidney injury, overall and stratified by age category and sex (complete case analysis). **Table S4**. Hazard ratio for all-cause readmission during 90-day periods after index hospitalization for acute exacerbation of chronic obstructive pulmonary disease, according to acute kidney injury, overall and stratified by age category and sex (complete case analysis). **Table S5**. Hazard ratio for all-cause readmission during 30-day and 90-day periods after index hospitalization for acute exacerbation of chronic obstructive pulmonary disease, according to acute kidney injury and acute kidney injury with dialysis. **Table S6**. The ten most frequent principal diagnoses of 90-day readmissions in patient hospitalized for acute exacerbation of chronic obstructive pulmonary disease, stratified by acute kidney injury.

## Data Availability

HCUP’s Nationwide and State-Specific Databases are available for purchase from the online HCUP distributor (information available at: https://www.hcup-us.ahrq.gov/tech_assist/centdist.jsp).

## Abbreviations

AECOPD: Acute exacerbation of chronic obstructive pulmonary disease
AHRQ: Agency for Healthcare Research and Quality
AKI: Acute kidney injury
CKD: Chronic kidney disease
COPD: Chronic obstructive pulmonary disease
HCUP: Healthcare Cost and Utilization Project
HRRP: Hospital Readmissions Reduction Program
ICD-9-CM: International Classification of Diseases, Ninth Revision, Clinical Modification
SID: State Inpatient Database
