# Supplementary figures and images for: Association of acute kidney injury with readmissions after hospitalization for acute exacerbation of chronic obstructive pulmonary disease: a population-based study
Source: BMC Nephrol. 2020 Apr 3;21:116. doi: 10.1186/s12882-020-01780-2 (PMC7119005; doi:10.1186/s12882-020-01780-2)

**Supplemental Figure 1.** **Patient flow of the study**


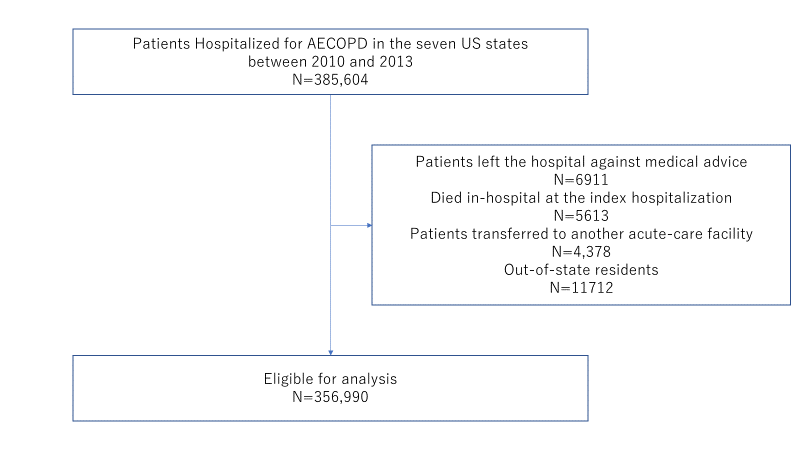

Supplement: Supplementary file 1 — Additional file 1 : Figure S1. Patient flow of the study. AECOPD, acute exacerbation of chronic obstructive pulmonary disease. [file 12882_2020_1780_MOESM1_ESM.docx]
