# Supplementary material for: Association of acute kidney injury with readmissions after hospitalization for acute exacerbation of chronic obstructive pulmonary disease: a population-based study
Source: BMC Nephrol. 2020 Apr 3;21:116. doi: 10.1186/s12882-020-01780-2 (PMC7119005; doi:10.1186/s12882-020-01780-2)
Supplement: Supplementary file 2 — Additional file 2 : Table S1. Characteristics of patients hospitalized for acute exacerbation of chronic obstructive pulmonary disease by readmission status. Table S2. Characteristics of patients hospitalized for acute exacerbation of chronic obstructive pulmonary disease by acute kidney injury (complete case analysis). Table S3. Hazard ratio for all-cause readmission during 30-day periods after index hospitalization for acute exacerbation of chronic obstructive pulmonary disease, according to acute kidney injury, overall and stratified by age category and sex (complete case analysis). Table S4. Hazard ratio for all-cause readmission during 90-day periods after index hospitalization for acute exacerbation of chronic obstructive pulmonary disease, according to acute kidney injury, overall and stratified by age category and sex (complete case analysis). Table S5. Hazard ratio for all-cause readmission during 30-day and 90-day periods after index hospitalization for acute exacerbation of chronic obstructive pulmonary disease, according to acute kidney injury and acute kidney injury with dialysis. Table S6. The ten most frequent principal diagnoses of 90-day readmissions in patient hospitalized for acute exacerbation of chronic obstructive pulmonary disease, stratified by acute kidney injury. [file 12882_2020_1780_MOESM2_ESM.docx]

**Supplemental Table 1. Characteristics of patients hospitalized for acute exacerbation of chronic obstructive pulmonary disease by readmission status**

|  | **30-day readmission** | **No readmission** |  |
| --- | --- | --- | --- |
| **Characteristics** | n=58,006 | n=298,375 | **P value** |
| Age (year), median (IQR) | 72 (62-81) | 70 (60-80) | <0.001 |
| Male | 32,418 (55.8) | 175,018 (58.6) | <0.001 |
| Race/ethnicity |  |  | <0.001 |
| Non-Hispanic white | 41,595 (73.6) | 2013,521 (74.0) |  |
| Non-Hispanic black | 6,340 (11.2) | 30,538 (10.6) |  |
| Hispanic | 6,211 (11.0) | 31,715 (11.0) |  |
| Others | 2,394 (4.2) | 12,857 (4.5) |  |
| Primary health insurance |  |  | <0.001 |
| Medicare | 44,140 (76.1) | 209,421 (70.1) |  |
| Medicaid | 6,806 (11.7) | 31,889 (10.7) |  |
| Private | 4,459 (7.7) | 36,167(12.1) |  |
| Others | 2,633 (4.6) | 21,158 (7.2) |  |
| Median household income quartile |  |  | <0.001 |
| 1 (lowest) | 18,326 (32.3) | 92,101 (31.6) |  |
| 2 | 15,327 (27.0) | 80,508 (27.6) |  |
| 3 | 13,170 (23.2) | 69,073 (23.7) |  |
| 4 (highest) | 9,846 (17.4) | 49,875 (17.1) |  |
| Patient residence |  |  | <0.001 |
| Metropolitan | 51,432 (88.6) | 259,955 (87.0) |  |
| Non-metropolitan | 6,644 (11.4) | 38,959 (13.0) |  |
| Selected comorbidities* |  |  |  |
| Congestive heart failure | 19,958 (33.7) | 69,888 (23.4) | <0.001 |
| Depression | 9,648 (16.6) | 46,182 (15.4) | <0.001 |
| Diabetes | 18,954 (32.6) | 85,365 (28.6) | <0.001 |
| Hypertension | 40,268 (69.3) | 199,253 (66.7) | <0.001 |
| Obesity | 9,071 (15.6) | 46,772 (15.6) | 0.86 |
| Peripheral artery disease | 5,413 (9.3) | 22,515 (7.5%) | <0.001 |
| Hospital length-of-stay |  |  | <0.001 |
| <3 days | 13,177 (22.7) | 88,299 (29.5) |  |
| 3-4 days | 18,984 (32.7) | 105,863 (35.4) |  |
| 5-6 days | 11,454 (19.7) | 54,476 (18.2) |  |
| ≥7 days | 14,461 (24.9) | 50,276 (16.9) |  |
| Hospital state |  |  | <0.001 |
| Arkansas | 2,494 (4.3) | 14,845 (5.0) |  |
| California | 11,700 (20.1) | 56,774 (19.0) |  |
| Florida | 23,432 (40.3) | 122,580 (41.0) |  |
| Iowa | 1,872 (3.6) | 10,717 (3.6) |  |
| Nebraska | 1,122 (1.9) | 6,666 (2.2) |  |
| New York | 16,962 (29.2) | 83,568 (28.0) |  |
| Utah | 494 (0.9) | 3,764 (1.3) |  |

Abbreviations: AKI, acute kidney injury; IQR, interquartile range

Data are shown as n (%) unless otherwise specified

* Selected from 29 Elixhauser comorbidity measures and arrhythmia

**Supplemental Table 2. Characteristics of patients hospitalized for acute exacerbation of chronic obstructive pulmonary disease by acute kidney injury (complete case analysis)**

|  | **AKI** | **Non-AKI** |  |
| --- | --- | --- | --- |
| **Characteristics** | n=23,366  (6.9%) | n=312,918  (93.1%) | **P value** |
| Age (year), median (IQR) | 76 (67-83) | 70 (60-80) | <0.001 |
| Male | 11,816 (50.6) | 128,141 (41.0) | <0.001 |
| Race/ethnicity |  |  | <0.001 |
| Non-Hispanic white | 16,961 (72.6) | 233,307 (74.6) |  |
| Non-Hispanic black | 2,763 (11.7) | 32,0460 (10.4) |  |
| Hispanic | 2,515 (10.8) | 34,188 (10.9) |  |
| Others | 1,154 (4.9) | 12,963 (4.1) |  |
| Primary health insurance |  |  | <0.001 |
| Medicare | 19,093 (81.7) | 220,911 (70.6) |  |
| Medicaid | 1,678 (7.2) | 33,0951 (10.6) |  |
| Private | 1,559 (6.7) | 36,554 (11.7) |  |
| Others | 1,036 (4.4) | 21,502 (6.9) |  |
| Median household income quartile |  |  | <0.001 |
| 1 (lowest) | 6,919 (29.6) | 99,678 (31.7) |  |
| 2 | 6,3037 (25.8) | 86,506 (27.6) |  |
| 3 | 5,696 (24.4) | 783,665 (23.5) |  |
| 4 (highest) | 4,714 (20.2) | 53,069 (17.0) |  |
| Patient residence |  |  | <0.001 |
| Metropolitan | 21,296 (91.1) | 274,440 (87.7) |  |
| Non-metropolitan | 2,070 (8.9) | 38,478 (12.3) |  |
| Selected comorbidities* |  |  |  |
| Congestive heart failure | 10,699 (45.8) | 73,880 (23.6) | <0.001 |
| Depression | 3,231 (13.8) | 50,4120 (16.0) | <0.001 |
| Diabetes | 9,879 (42.3) | 88,573 (28.3) | <0.001 |
| Hypertension | 19,148 (81.9) | 207,865 (66.4) | <0.001 |
| Obesity | 4,846 (20.7) | 48,369 (15.5) | <0.001 |
| Peripheral artery disease | 2,874 (12.3) | 23,927 (7.6) | <0.001 |
| Hospital length-of-stay |  |  | <0.001 |
| <3 days | 3,346 (14.3) | 91,356 (29.2) |  |
| 3-4 days | 6,752 (28.9) | 110,813 (35.4) |  |
| 5-6 days | 5,084 (21.8) | 57,366 (18.2) |  |
| ≥7 days | 8,184 (35.0) | 53,383 (17.1) |  |
| Hospital state |  |  | <0.001 |
| Arkansas | 1,515 (6.3) | 15,158 (4.8) |  |
| California | 4,333 (18.5) | 60,855 (19.4) |  |
| Florida | 10,139 (43.4) | 133,083 (42.5) |  |
| Iowa | 736 (3.1) | 11,328 (3.6) |  |
| New York | 6,378 (27.3) | 89,233 (28.5) |  |
| Utah | 265 (1.0) | 3,261 (1.0) |  |

Abbreviations: AKI, acute kidney injury; IQR, interquartile range

Data are shown as n (%) unless otherwise specified

* Selected from 29 Elixhauser comorbidity measures and arrhythmia

**Supplemental Table 3. Hazard ratio for all-cause readmission during 30-day periods after index hospitalization for acute exacerbation of chronic obstructive pulmonary disease, according to acute kidney injury, overall and stratified by age category and sex (complete case analysis)**

|  | **30-day readmission rate** | | **Unadjusted model** | | **Adjusted model*** | |
| --- | --- | --- | --- | --- | --- | --- |
|  | AKI  (n=23,366) | Non-AKI  (n=312,918) | HR (95% CI) | P-value | HR (95% CI) | P-value |
| **Overall** (n=336,284) | 22.6% | 15.9% | 1.55 (1.50-1.60) | <0.001 | 1.06 (1.03-1.10) | 0.001 |
| **Age category** |  |  |  |  |  |  |
| 40-64 years (n=114,486) | 21.9% | 14.1% | 1.63 (1.51-1.76) | <0.001 | 1.03 (0.95-1.11) | 0.51 |
| ≥65 years (n=221,798) | 22.7% | 16.8% | 1.47 (1.42-1.53) | <0.001 | 1.08 (1.03-1.12) | <0.001 |
| **Sex** |  |  |  |  |  |  |
| Men (n=139,957) | 22.2% | 16.9% | 1.42 (1.35-1.48) | <0.001 | 1.03 (0.98-1.08) | 0.29 |
| Women (n=196,327) | 23.1% | 15.3% | 1.66 (1.59-1.74) | <0.001 | 1.10 (1.05-1.16) | <0.001 |

Abbreviations: AKI, acute kidney injury; HR, hazard ratio; CI, confidence interval

* Cox proportional hazards model adjusting for age, sex, race/ethnicity, insurance status, estimated household income, residential status, hospital length-of-stay, hospital state, and Elixhauser comorbidity measures and arrhythmia with generalized estimating equations to account for patient clustering within hospitals

**Supplemental Table 4 Hazard ratio for all-cause readmission during 90-day periods after index hospitalization for acute exacerbation of chronic obstructive pulmonary disease, according to acute kidney injury, overall and stratified by age category and sex (complete case analysis)**

|  | **90-day readmission rate** | | **Unadjusted model** | | **Adjusted model*** | |
| --- | --- | --- | --- | --- | --- | --- |
|  | AKI  (n=23,366) | Non-AKI  (n=312,918) | HR (95% CI) | P-value | HR (95% CI) | P-value |
| **Overall** (n=336,284) | 40.0% | 31.2% | 1.47 (1.43-1.51) | <0.001 | 1.03 (1.00-1.05) | 0.04 |
| **Age category** |  |  |  |  |  |  |
| 40-64 years (n=114,486) | 38.2% | 27.9% | 1.59 (1.50-1.69) | <0.001 | 0.99 (0.94-1.06) | 0.79 |
| ≥65 years (n=221,798) | 40.5% | 32.9% | 1.38 (1.34-1.43) | <0.001 | 1.02 (0.98-1.05) | 0.13 |
| **Sex** |  |  |  |  |  |  |
| Men (n=139,957) | 39.2% | 32.4% | 1.35 (1.30-1.40) | <0.001 | 0.98 (0.93-1.02) | 0.26 |
| Women (n=196,327) | 40.8% | 30.3% | 1.58 (1.53-1.64) | <0.001 | 1.04 (0.99-1.08) | 0.07 |

Abbreviations: AKI, acute kidney injury; HR, hazard ratio; CI, confidence interval

* Cox proportional hazards model adjusting age, sex, race/ethnicity, insurance status, estimated household income, residential status, hospital length-of-stay, hospital state, and Elixhauser comorbidity measures and arrhythmia with generalized estimating equations to account for patient clustering within hospitals

**Supplemental Table 5 Hazard ratio for all-cause readmission during 30-day and 90-day periods after index hospitalization for acute exacerbation of chronic obstructive pulmonary disease, according to acute kidney injury and acute kidney injury with dialysis**

|  | **Readmission rate**  **n (%)** | **Unadjusted model** | | **Adjusted model*** | |
| --- | --- | --- | --- | --- | --- |
|  |  | HR (95% CI) | P-value | HR (95% CI) | P-value |
| **30-day readmission** |  |  |  |  |  |
| No AKI (n=332,157) | 52,524 (15.8%) | Reference | - | Reference | - |
| AKI without dialysis (n=23,465) | 5,248 (22.5%) | 1.47 (1.43-1.51) | <0.001 | 1.06 (1.03-1.10) | 0.001 |
| AKI with dialysis (n=1,368) | 268 (19.6%) | 1.30 (1.13-1.48) | <0.001 | 1.03 (0.90-1.20) | 0.67 |
| **90-day readmission** |  |  |  |  |  |
| No AKI (n=332,157) | 103,067 (31.0%) | Reference | - | Reference | - |
| AKI without dialysis (n=23,465) | 9,329 (39.8%) | 1.43 (1.36-1.51) | <0.001 | 1.03 (0.99-1.06) | 0.06 |
| AKI with dialysis (n=1,368) | 521 (38.1%) | 1.35 (1.23-1.53) | <0.001 | 0.98 (0.87-1.05) | 0.87 |

Abbreviations: AKI, acute kidney injury; HR, hazard ratio; CI, confidence interval

* Cox proportional hazards model adjusting age, sex, race/ethnicity, insurance status, estimated household income, residential status, hospital length-of-stay, hospital state, and Elixhauser comorbidity measures and arrhythmia with generalized estimating equations to account for patient clustering within hospitals

**Supplemental Table 6. The ten most frequent principal diagnoses of 90-day readmissions in patient hospitalized for acute exacerbation of chronic obstructive pulmonary disease, stratified by acute kidney injury**

| **Without AKI (n=98,983)** | | **With AKI (n=13,934)** | |
| --- | --- | --- | --- |
| **Primary diagnosis*** | **n (%)** | **Primary diagnosis*** | **n (%)** |
| COPD and bronchiectasis | 25,031 (25.3) | Septicemia | 1,850 (13.3) |
| Pneumonia | 7,165 (7.2) | COPD and bronchiectasis | 1,700 (12.2) |
| Asthma | 6,834 (6.9) | Acute renal failure | 1,573 (11.3) |
| Respiratory failure | 6,470 (6.5) | Congestive heart failure | 1,263 (9.1) |
| Congestive heart failure | 5,636 (5.7) | Respiratory failure | 1,031 (7.4) |
| Septicemia | 3,007 (3.0) | Pneumonia | 933 (6.7) |
| Rehabilitation care | 2,547 (2.6) | Asthma | 370 (2.7) |
| Cardiac dysrhythmias | 2,376 (2.4) | Acute myocardial infarction | 263 (1.9) |
| Nonspecific chest pain | 1,665 (1.7) | Cardiac dysrhythmias | 259 (1.9) |
| Skin and subcutaneous tissue infections | 1,337 (1.4) | Intestinal infection | 221 (1.6) |

*The primary diagnoses (>14,000 *ICD-9-CM* diagnosis codes) consolidated into 285 mutually exclusive diagnostic categories by using the AHRQ-defined *Clinical Classifications Software.*
